# Students’ Views towards Sars-Cov-2 Mass Asymptomatic Testing, Social Distancing and Self-Isolation in a University Setting during the COVID-19 Pandemic: A Qualitative Study

**DOI:** 10.3390/ijerph18084182

**Published:** 2021-04-15

**Authors:** Holly Blake, Holly Knight, Ru Jia, Jessica Corner, Joanne R. Morling, Chris Denning, Jonathan K. Ball, Kirsty Bolton, Grazziela Figueredo, David E. Morris, Patrick Tighe, Armando Mendez Villalon, Kieran Ayling, Kavita Vedhara

**Affiliations:** 1 School of Health Sciences, University of Nottingham, Nottingham NG7 2HA, UK; 2 NIHR Nottingham Biomedical Research Centre, Nottingham NG7 2UH, UK; joanne.morling@nottingham.ac.uk; 3 School of Medicine, University of Nottingham, Nottingham NG7 2UH, UK; holly.knight@nottingham.ac.uk (H.K.); ru.jia@nottingham.ac.uk (R.J.); kieran.ayling@nottingham.ac.uk (K.A.); kavita.vedhara@nottingham.ac.uk (K.V.); 4 University Executive Board, University of Nottingham, Nottingham NG7 2RD, UK; jessica.corner@nottingham.ac.uk; 5 Biodiscovery Institute, School of Medicine, University of Nottingham, Nottingham NG7 2RD, UK; chris.denning@nottingham.ac.uk (C.D.); jonathan.ball@nottingham.ac.uk (J.K.B.); 6 School of Life Sciences, University of Nottingham, Nottingham NG7 2RD, UK; paddy.tighe@nottingham.ac.uk; 7 School of Mathematical Sciences, University of Nottingham, Nottingham NG7 2RD, UK; kirsty.bolton@nottingham.ac.uk; 8 School of Computer Sciences, University of Nottingham, Nottingham NG8 1BB, UK; g.figueredo@nottingham.ac.uk; 9 Faculty of Engineering, University of Nottingham, Nottingham NG7 2RD, UK; david.morris@nottingham.ac.uk (D.E.M.); armando.mendez@nottingham.ac.uk (A.M.V.)

**Keywords:** COVID-19, SARS-CoV-2, coronavirus, mass testing, social isolation, social distancing, mental health, students, focus groups, qualitative

## Abstract

We aimed to explore university students’ perceptions and experiences of SARS-CoV-2 mass asymptomatic testing, social distancing and self-isolation, during the COVID-19 pandemic. This qualitative study comprised of four rapid online focus groups conducted at a higher education institution in England, during high alert (tier 2) national COVID-19 restrictions. Participants were purposively sampled university students (*n =* 25) representing a range of gender, age, living circumstances (on/off campus), and SARS-CoV-2 testing/self-isolation experiences. Data were analysed using an inductive thematic approach. Six themes with 16 sub-themes emerged from the analysis of the qualitative data: ‘Term-time Experiences’, ‘Risk Perception and Worry’, ‘Engagement in Protective Behaviours’, ‘Openness to Testing’, ‘Barriers to Testing’ and ‘General Wellbeing’. Students described feeling safe on campus, believed most of their peers are adherent to protective behaviours and were positive towards asymptomatic testing in university settings. University communications about COVID-19 testing and social behaviours need to be timely and presented in a more inclusive way to reach groups of students who currently feel marginalised. Barriers to engagement with SARS-CoV-2 testing, social distancing and self-isolation were primarily associated with fear of the mental health impacts of self-isolation, including worry about how they will cope, high anxiety, low mood, guilt relating to impact on others and loneliness. Loneliness in students could be mitigated through increased intra-university communications and a focus on establishment of low COVID-risk social activities to help students build and enhance their social support networks. These findings are particularly pertinent in the context of mass asymptomatic testing programmes being implemented in educational settings and high numbers of students being required to self-isolate. Universities need to determine the support needs of students during self-isolation and prepare for the long-term impacts of the pandemic on student mental health and welfare support services.

## 1. Introduction

Coronavirus disease 2019 (COVID-19) is caused by the severe acute respiratory syndrome coronavirus 2 (SARS-CoV-2). The World Health Organization declared the outbreak of coronavirus disease (COVID-19) a pandemic in March 2020. During this time, restrictions on movement were put into place worldwide, to flatten the curve of infection through social distancing. The functioning of colleges and universities during the pandemic presents a challenge. Globally, strategies to manage the situation have included containment and mitigation, such as access control with contact tracing and quarantine, hygiene, sanitation, ventilation, and social distancing. In the United Kingdom (UK), this required rapid development of local organisational COVID-19 policies in universities, requiring regular adaptation in line with evolving updates from the UK Higher Education Taskforce, and rapid changes in government policy and guidance, as the national situation changes. In the UK, universities rapidly transitioned to online teaching and learning during the first surge of COVID-19 in March 2020, followed by large-scale reopening of campuses for the new academic year in September/October 2020. This mass movement of students from across the UK and overseas aligned with a second surge of COVID-19 across the UK [1] and the establishment of a national tiered system of restrictions to address local outbreaks of COVID-19 (Supplementary File S1). 

The proportion of asymptomatic infection among COVID-19 positive persons is found to be high, with a substantial transmission potential [2]. A systematic review found that in two general population studies, the proportion of asymptomatic COVID-19 infection at time of testing was 20% and 75%, respectively, and among three studies in contacts it was 8.2% to 50% [2]. In the absence of a national strategy or policy, some universities developed local capability for frequent and regular mass asymptomatic SARS-CoV-2 testing programmes [3,4] in effort to reduce the risks [5] of viral transmission between asymptomatic students. This approach aimed to maximise the safety of staff, students, and local communities, and aligned with recommendations made by the UK’s Independent SAGE Behavioural Advisory Group [6]. Without national guidance, there was hesitancy around asymptomatic testing, as the implications for students’ social behaviours and wellbeing were unknown.

The success of mass testing approaches relies on high levels of testing and social isolation [7,8] to reduce viral transmission. Further, a combination of moderate physical distancing measures, self-isolation, and contact tracing is more likely to achieve control of severe virus transmission [7]. However, we know little about students’ views towards these approaches to mitigation and containment. Although adherence to COVID-19 social regulations was generally high in the UK population (>90%), less than half of the population adhered to full self-isolation (duration adjusted adherence to full self-isolation was 42.5%) [9]. Additionally, 46% of ‘resisters’ to the lockdown rules were from younger age groups (16–24 years) [10]. Since 1 in 3 people aged 18 to 24 years were in full time education [11] it is possible that education settings host a high proportion of individuals who are less likely to adhere to social isolation. There is a high prevalence of younger age groups in universities; in 2019/20 there were 2.46 million students at UK higher education institutions [12], with 18–19 year-olds making the largest contribution to the Higher Education Initial Participation (HEIP) measure (the sum of the initial entry percentages in each of the age groups from 17 to 30 years, in a given academic year) [13].

This study was conducted at a university in England, in October 2020, at the beginning of the Autumn term, at the time of a second surge of COVID-19 in the UK. Earlier in the year (April–October 2020), a pilot asymptomatic testing programme was implemented at the same institution with a high reported acceptability of SARS-CoV-2 asymptomatic testing and logistics (virus—swab and saliva; antibody—finger prick), and a high willingness to engage in future testing (94.9%) [4]. Self-reported adherence to weekly virus testing in this pilot delivery was high (92.4% completed ≥6 tests; 70.8% submitted all 10 swabs; 89.2% completed ≥1 saliva sample) and 76.9% submitted ≥3 blood samples [4]. Although there was a paucity of evaluations, high uptake of asymptomatic testing was also demonstrated at another UK institution [3,14].

However, at our institution, at the start of the Autumn term, there was a wide-scale deployment of local asymptomatic testing in residential student halls with much lower uptake than was found in the pilot service. Uptake amongst students first offered an asymptomatic test in residence-based deployments was 13% up to the end of October. During October there was a marked decrease in testing uptake, beginning at 58% in the first deployment in early October and decreasing to as low as 5% in a late-October deployment. Concurrently during this time there were around 2000 self-reports of positive SARS-CoV-2 tests in students, the majority of which were associated with symptomatic infection. A significant minority of these reports were associated with students following advice to seek confirmatory UK National Health Service (NHS) community testing, in response to a positive test identified through the University’s asymptomatic testing service. The institution had one of the highest reported rates of COVID-19 in the country at that time [15], although it was anticipated that the asymptomatic testing programme would identify cases earlier and more quickly as it rolled out through the term and detected positive cases that might have otherwise remained undetected. The testing service was intended to reduce asymptomatic transmission and the number of future cases. Nevertheless, the impact of COVID-19 on social isolation at our institution was dramatic during this time, with many more students reporting entering isolation than reporting positive tests. Despite publication of isolation numbers being uncommon, the total number of people self-isolating across 45 universities with positive cases reported to be above 3540 within just 9 weeks [16], suggesting that this was a common experience across UK universities. The overall aim of the study was therefore to explore university students’ perceptions and experiences of SARS-CoV-2 asymptomatic testing and strategies for mitigation (social distancing) and containment (self-isolation) in a higher education setting. The findings provide insight into students’ barriers to testing uptake and adherence to social restrictions, contribute to a wider debate around mass testing approaches in a pandemic [17,18,19,20], and the impact of mitigation and containment strategies on young people’s social behaviours and wellbeing.

## 2. Methods

### 2.1. Study Design

This was a qualitative focus group study involving four online focus groups with a total of 25 participants undertaken in a two-week period, during October 2020. The study design adhered to the consolidated criteria for reporting qualitative studies (COREQ) guidelines [21] (Supplementary File S2). The research protocol was approved by the University of Nottingham Faculty of Medicine and Health Sciences Research Ethics Committee (Ref: FMHS 76-0920).

### 2.2. Study Context

During this time, England was subject to national coronavirus restrictions. The participating university was in a region categorised as ‘tier 2 high alert’, during which government restrictions prevented people from meeting indoors with individuals or groups from outside of their household or support bubble. At this time, people were advised that no households should mix indoors or in groups of more than 6 outdoors with social distancing, remote working (and studying), other restrictions on travel, facilities, and services (Supplementary File S1). Students had to contend with abrupt changes in the way that education was delivered, the risks of COVID-19 more broadly, significant reductions in social contact, and separation from friends and family due to social distancing measures. Large numbers of students had to adapt to confinement strategies in residential education settings, including shared student accommodation and houses in multiple occupation. Due to increasing numbers of positive cases locally and nationally, many students were required to self-isolate during this time, which meant staying in their home or place of residence and not going outside for any reason, including not travelling to a different place of residence. At the time of data collection, a mass asymptomatic SARS-CoV-2 testing programme was underway at the participating university [4], with testing deployments taking place in a small number of university halls of residence, with plans for a rapid roll out of testing to all university staff and students being developed. 

### 2.3. Participants, Sampling, and Recruitment

Participants were university students recruited from a single higher education institution via an established cohort study of students living on and off campus [22]. Purposive sampling was used to provide a diverse range of ages, genders, living circumstances (on/off campus), SARS-CoV-2 testing, and self-isolation experiences (Table 1 and Supplementary File S3). Students required to self-isolate were those that tested positive for SARS-CoV-2, lived with someone who had symptoms or had tested positive, or were identified as a contact of someone who had tested positive by the NHS ‘Test and Trace’. Of the 25 students in the sample, 12 were tested, of which 11 were symptomatic and one was asymptomatic. All participants were currently residing in the UK and gave informed consent online to be approached for interview via Jisc Online Surveys, and additional verbal consent was provided and audio-recorded prior to the start of the focus group. Recruitment continued until achievement of maximum variation sampling, in terms of the pre-specified interviewee characteristics. The 2-week data collection period allowed for rapid data analysis so that findings of the study could feed into university COVID-19 strategy around mass testing and student support. Students were not compensated for their participation. Online data collection was necessary due to social isolation policy. However, online focus groups are commonly used in health research to capitalise on group interaction in diverse and geographically dispersed participants, to collect rich responses to questions posed in a cost saving and convenient way [23].

### 2.4. Online Focus Groups

Students took part in one of four focus groups (*n* = 3–11 in each group) held online using video-conferencing facilities. The focus groups lasted for 58 to 70 min (mean = 64 min). Two psychologists (H.B./H.K.) generated the question guide, moderated the focus groups and analysed the data. We used a semi-structured question guide focused on the research outcomes of several studies [4,22,24], to cover the main topics related to the outcomes of the study. A draft topic guide was developed by the same two psychologists (H.B./H.K.). To judge the relevance of the topics and the possible emotional impact on students, the topic guide was discussed with experts who had expertise in mass SARS-CoV-2 testing (*n* = 2), psychology (*n* = 2), university operations (*n* = 2), student wellbeing (*n* = 3) and student members of a Patient and Public Involvement and Engagement (PPIE) group (*n* = 2). Following feedback, the draft topic guide was pilot tested for comprehensibility and level of burden with two students who were not participants in the study, which resulted in minor amendments to question wording. Both moderators were trained in qualitative research and interview skills and were not involved in delivery of the asymptomatic SARS-CoV-2 testing programme. Focus groups were conducted according to recommendations from NHS England’s focus group guide [25]. A funnel approach was used with broader, generic questions at the outset (e.g., introductions), leading to more directed questioning (e.g., views on specific issues). The purpose was to build rapport within the group and ensure there was enough ‘lead-in’ time for participants to feel comfortable about contributing to the discussion. Due to the nature of the discussion, and the timing of data collection amidst a surge in the pandemic, it was not deemed appropriate to require all students to respond to every question, although every effort was made to encourage participation within the group. All focus groups followed the same questioning route (Supplementary File S4), were audio-recorded and transcribed verbatim. Participants are referred to as new students (first year students beginning their studies in the Autumn term) and returning students (those resuming their studies in the Autumn term following a summer break).

### 2.5. Data Analysis

The audio recordings were professionally transcribed. Two experienced researchers independently familiarised themselves with the data (H.K./H.B.). We performed inductive thematic analysis [26]. Data were examined for patterns and recurrent instances, which were then systematically identified across the dataset. Due to the rapidity of the study, one researcher (H.K.) coded all focus groups transcripts using open coding [27] into codes and subcodes. To ensure reliability of data interpretations, two researchers (H.K./H.B.) then independently read the emerging codes and supporting quotations to enhance the accountability of the analysis [26]. Codes were individually and critically examined by both researchers, and the overlapping codes and subcodes were further refined and grouped together. Codes and subcodes with similar characteristics were then grouped into meaningful overarching themes that emerged organically from the data. Themes, codes and subcodes were confirmed by two student participants. Given the aim of the study, the sample specificity, the rich dataset, in-depth insights into the phenomena of interest and the analysis approach adopted, the qualitative sample was deemed to have sufficient information power [28].

## 3. Results

Six themes emerged from the analysis of the qualitative data from the focus groups—‘Term-time Experiences’, ‘Risk Perception and Worry’, ‘Engagement in Protective Behaviours’, ‘Openness to Testing’, ‘Barriers to Testing’, and ‘General Wellbeing’. A thematic map illustrating the relationships between the key themes and subthemes is provided in Supplementary File S5. Table 2 shows the list of all key themes and subthemes and the representative quotes, together with the frequency (and %) of students contributing independent statements of agreement within each subtheme.

## 4. Discussion

This study explored university students’ perceptions and experiences of university life during COVID-19, SARS-CoV-2 mass testing, and strategies for mitigation (social distancing) and containment (self-isolation) of the virus, during the second surge of the COVID-19 pandemic in the UK, with six emerging themes. Theme 1 (‘Term-time Experiences’) highlights the impacts of COVID-19 on practical issues surrounding students’ daily life and academic studies, alongside university approaches to protect and safeguard. Themes 2 and 3 (‘Risk Perception and Worry’; ‘Engagement in Protective Behaviours’) demonstrate the individual and structural drivers of students’ engagement with social behaviours that protect against virus transmission. Themes 4 and 5 (‘Openness to Testing’, ‘Barriers to Testing’) highlight students’ openness to mass asymptomatic testing alongside the barriers and enablers of testing and its consequences. Theme 6 (‘General Wellbeing’) highlights the broader impacts of the pandemic on social and mental wellbeing, which are core concepts interwoven within Themes 1–6.

### 4.1. Impacts on University Life during a Pandemic

We found that COVID-19 impacted significantly on student experience of university life. It is clear that students in university-managed accommodation experienced some practical complications in accessing basic supplies at the start of the term, and although these issues likely contributed to student anxiety, they were temporary and quickly resolved at a local level. Nevertheless, there were students for whom access to food and basic supplies was likely to be more challenging during this time (e.g., students living off-campus in privately owned accommodation, particularly international students arriving to the UK for the first time). These groups might be at particular risk since food insecurity (worry about how, and where, to access food) was identified in 35% of students during COVID-19 lockdown, and students’ living arrangements during the pandemic was found to be the strongest predictor of food insecurity [29].

The University’s approach to safeguarding students while managing the continuation of studies was well received, although the pandemic had dramatically impacted the social aspects of learning and university life. Many students reported that the university had provided sufficient cleaning equipment and safety measures to make students feel comfortable on campus. This applied across different campus settings, including accommodation, libraries, lecture halls and gym facilities. Although perceptions of safety on campus were high, some felt that the safe-guarding measures negatively impacted the broader student experience. In particular, individuals undertaking laboratory-based research reported that their studies were heavily impacted by university-wide safe-guarding building closures. To some, the safe-guarding processes surrounding self-isolation in halls of residence were viewed to be particularly restrictive. However, this was mitigated by regular communications from university staff that improved students’ experience of university life, and appeared to enhance students’ feelings of connectedness, particularly during periods of social isolation.

The crisis-response migration of universities to online education early in the pandemic was essential and enabled the continuation of education in universities at that time [30] but the transition was not without its impacts. Impacts on studies were particularly notable at the start of the Autumn term alongside efforts to shift to online teaching and learning, and to mobilize mitigation and containment strategies in a short period. Students endorsed varying levels of adjustment to learning online. Many students described adapting well to online functioning, noting that the course conveners had also adapted well to this shift, and consequently, their education did not suffer. Some students enjoyed the novelty of self-guided learning. However, multiple students found this format to be disorganized at the outset, with classes cancelled or rescheduled at the last minute at the start of the Autumn term. The transition to unfamiliar online learning environments was particularly challenging for new students who had not yet established their friendship groups and international students for whom English was not their native language. It was also noted that the move to online learning resulted in the loss of potential networking opportunities that might have arisen through the course if it were delivered face-to-face. The immediate challenges for higher education institutions were apparent, with regards to access to technical infrastructure, pedagogies for distance learning, competences (of students and staff), and managing the requirements of specific fields of study (e.g., hands-on learning requirements, field work, assessments) [31]. This rapid transition to online teaching and learning might precipitate enhanced teaching and learning opportunities in the future [32], by increasing opportunities for flexible learning approaches [31]. However, the requirement to adapt at speed to unfamiliar online e-learning approaches in the context of the pandemic is challenging for some.

For students in our study, many had adapted well to online learning, despite the early hitches of the transition period. While students generally felt that the university had appropriately managed safeguarding, the combined impact of safeguarding and the transition to online learning had limited opportunities for important social contact. Students who seemed to fare better were those who had received more regular contacts from university staff during the pandemic, and particularly through periods of self-isolation. As a result, universities should act on generating opportunities for social support and networking, which could be delivered through academic departments, sports, wellbeing facilities, clubs and societies.

### 4.2. Risk Perceptions, Adherence and Social Behaviours

With regards COVID-19 mitigation, students in our study were highly conscious of the risks of COVID-19, although many who considered themselves to be in good health were more concerned with the asymptomatic spread of COVID-19 to others than the risk of contracting the virus themselves. Previous experience with COVID-19 also heightened students’ fears about the impact of the virus on others, particularly those in vulnerable groups (e.g., older relatives). However, those with a pre-existing health condition they felt put them at increased risk, conveyed a strong concern about the potential impact of contracting COVID-19 themselves. Students with pre-existing health conditions described concern about going into public settings for fear that others might put them at risk.

In our sample, there were two factors that were perceived to reduce compliance with social distancing in a minority of students and this did not seem to be related to risk perception, but more to the environment and desire for social contact. First, some of the residences and educational buildings had narrow corridors and ‘bottlenecks’, preventing the 2-m distancing between people that is required by UK government restrictions, which was seen to present an environmental constraint. Second, some students had an overwhelming desire to socialize that meant they were non-compliant with peers, despite adhering to social distancing in other contexts (with strangers). However, students perceived that only a small minority of the general student body were non-adherent to social distancing.

Although some improvement occurred over time, levels of adherence to test, trace, and isolate are low in the UK [10]. Our participants suggested that adherence to self-isolation might be more likely in students who experienced COVID-19 symptoms than in those who were self-isolating for other reasons. This might be due to greater perceptions of risk and disease severity in those who had personal experience of COVID-19 (e.g., [22]), and that people with high risk perception around infectious diseases tend to take preventive behaviour [33]. However, risk perceptions can only partially explain this, since adherence to self-isolation in young people is strongly related to structural vulnerabilities and availability of resources (e.g., social support with food access and caregiving responsibilities, financial hardship, and space in living accommodation) [34].

Students’ concerns about passing on COVID-19 to vulnerable loved ones indicates that adherence of university students to COVID-19 protective behaviours might be associated with a sense of social responsibility, and this was also identified in other populations of young people [35,36]. Although adherence to social distancing and protective behaviours was found to be lower in younger adults than other age groups [37], students in our study reported adhering to protective behaviours and observing compliance across the university more broadly. Nonetheless, they reported seeing or hearing that a minority of students were non-compliant with social distancing behaviours or self-isolation. This echoes data from the UK Office for National Statistics (ONS) Student Covid Insights Survey (SCIS) [38], which found that that 9 out of 10 university students reported complying with social distancing around the time of the study and were more likely to avoid leaving their accommodation completely than the general public, although the non-adherent minority were more likely to be from younger age groups [11].

Some studies indicated that non-compliance with public health advice during COVID-19 is associated with weaker feelings of moral obligation, low trust in authorities and individual characteristics related to antisocial potential [39]. Alternatively, it might be that non-compliant students simply perceive being around their peers, particularly in a campus environment and shared living accommodation, to be low risk, due to their familiarity with each other, and so the concept of social responsibility might feel less relevant to some individuals in this context. This could partially explain the high prevalence of COVID-19 outbreaks on university campuses across the UK.

Higher education providers are encouraged by the government to consider incentives for compliance, and disincentives for non-compliance including, in serious cases, the use of disciplinary measures [40]. For those willingly or repeatedly breaching University or Government guidance, policies, or laws in relation to COVID-19, there might, for example, be disciplinary investigations, fines, temporary or permanent withdrawal of students from university activities or a course of study, or referral to Police, Public Health, or Border Agencies. Nevertheless, a deeper understanding of the structural, psychological and social barriers to adherence might help reduce the occurrence of regulation breaches and non-compliance. Overall, social interaction is an integral part of students’ lives. Universities and colleges should consider the social impact of protective behaviours and offer social outlets for students when appropriate (e.g., providing opportunity for monitored socializing outdoors when it is safe to do so). Given the highlighted structural difficulties some students experienced with their accommodation providers, the university should set out clear guidance for both students and providers on practical, social, and emotional supports for students, on return to campus following national lockdown and during periods of self-isolation. These strategies might improve adherence to self-isolation and reduce fear of self-isolation, which might equally enhance uptake of testing. 

#### 4.2.1. Communications and Social Behaviours

Our study suggests that students on the whole are predominantly adherent to protective behaviours, but reduced compliance with social distancing and self-isolation guidance was also associated with perceived inadequacies in university communications at the time of the study, which were not always seen to be timely. Students reported that they were most likely to comply with guidance if it was presented in a simple format, supported by elucidation of the reasons behind the guidance. Students noted that communications from both the government and university were text heavy, and difficult to read and comprehend, particularly for international students. Students thought communications could be improved through the use of infographics rather than text. Multiple students reported that university communications were also relatively slow, meaning that students had already engaged in activities that could have caused spread of virus (e.g., use of communal space). Some students also reported finding it difficult to follow guidance, as a result of gaps in the government and university information pertinent to them.

However, students in this study recognized the challenges associated with communicating with large numbers of people in frequently changing national and global circumstances. Similarly, previous research conducted at the same institution found that most student participants were largely satisfied with university communications, with dissatisfaction expressed by a minority that was specifically related to an early approach to communicating negative test results at this institution, which was subsequently changed in response to student preference [4]. 

Government guidance emphasized that higher education providers should ensure that the rationale for protective behaviours is understood via clear and consistent messaging, while not assuming that everyone understands official guidelines [40]. We propose that institutional communications around COVID-19 might need to be more accessible and inclusive, since messaging at the time of the study was not universally understood amongst students, and the needs of certain groups (e.g., postgraduate students, international students, off-campus students) were not being met. It is important to consider these findings in the context of a fast moving and uncertain crisis situation, during which institutional COVID-19 strategies had to be developed and operationalized at speed. This required high responsivity to changes in local and national guidelines and procedures, with rapid communication of changes to university staff and students. It was advocated that organisational communications during the COVID-19 crisis should be succinct, to be read and understood [41]. Our findings might highlight a tension between the need for simplicity and readability of communications by the target audience, particularly students for whom English was not their first language. Additionally, the importance of communications (e.g., clarity, inclusion, and timeliness) in maximizing adherence to protective behaviours should not be underestimated. Given the identified link between desire for social contact and adherence to protective behaviours, messaging should emphasize the desirability of adhering to public health protocols, and signpost activities that minimize the boredom of self-isolation and maximize opportunities for social contact and activity engagement (e.g., virtual social interactions, exercise classes) [42].

#### 4.2.2. Communication Approaches

Given the high proportion of young people in universities, COVID-19 information provided to young people should be clear, delivered by a trusted source, should avoid giving visibility to non-adherence, and should promote positive behaviours to enact, rather than avoid negative behaviours [34]. Ideally, messages for students would be co-created with students [43], since it is well-established that young people are often more heavily influenced by their peers than by other age groups and are more likely to heed advice from those in similar age groups. Thus, ‘using the young person’s voice’ to deliver messaging would be helpful to reach higher education students in younger age groups. As ‘social influence agents’ who support or undermine health-related behaviours [44], peers both model, and influence, healthy and unhealthy behaviours [45]. Therefore, communications could emphasize social norms related to adherence to protective behaviours (e.g., what peers think, what peers do) [34]. Since young people in particular are generally more oriented towards short-term rewards rather than long-term consequences [46], messaging could emphasize the immediate impacts of COVID-19 such as the risks to loved ones, and young people should be thanked for their contribution to reduction of virus transmission. Communications should not just instruct young people on what to do but should include clear guidelines on how to enact protective behaviours (e.g., how to socialize in a COVID-safe way, how to socially distance in specific situations, and how to engage with peers who are non-adherent) [34].

### 4.3. Students and COVID Testing

Our study suggests that students at this institution remained positive towards the availability of local asymptomatic testing for SARS-CoV-2 and generally felt safe on the university campus at the time of the study (high alert, UK second surge) with mass testing in place, and during a time when the national situation had dramatically changed, and cases were rising [1]. With regards to the practicalities of testing, no particular problems were raised relating to any of the testing processes or procedures (NHS symptomatic community test—throat swab; University asymptomatic test—saliva). Some students reported that the throat swab test was uncomfortable, yet prior work suggests that students did not raise this as a barrier to the uptake of testing [4]. Studies in other populations suggest that discomfort was relatively low in both throat and nasal swabs, although nasal swabs were less likely to induce nausea or vomiting [47]. There is little published evidence in this area, although unpublished work suggests that saliva tests are a less intrusive approach with university students as compared to nasal swabs [48]. Testing uptake and self-isolation adherence could be low in education settings (e.g., [36]). Greater student adherence to SARS-CoV-2 asymptomatic testing is associated with their level of satisfaction with university communications [4]. Intrusiveness and convenience of testing procedures should also be considered and balanced alongside test sensitivity, to maximize testing uptake. Overall, our study showed that the availability of testing was seen by students to be an important approach to ‘getting control’ of the virus, although engagement with testing was more likely to be related to the emotional impacts of self-isolation and its consequences. To maximize uptake of asymptomatic testing, there needs to be significant support in place to manage the impacts of self-isolation on students’ social relationships and mental wellbeing. Further, the risk of unintended behavioural consequences of mass testing could not be dismissed, since our findings suggest that for a minority, a negative test result might instill a sense of false security and perceived immunity to COVID-19.

### 4.4. General Wellbeing and Mental Health

Overall, the long-lasting pandemic situation and associated restrictions have had psychological consequences in the general population [49], with young adults being particularly at risk for mental ill-health [22,24]. Specifically, in university students, mental health concerns were identified globally during the pandemic, with high rates of stress, anxiety, depression, and evidence of clinically relevant post-traumatic stress disorder [4,22,50,51,52].

Confinement strategies associated with COVID-19 were unavoidable during the COVID-19 pandemic but were shown to impact mental health and exacerbate social inequalities in university students [53]. Our study suggests that mental health plays a key role in students’ behavioural decision-making about engagement in protective behaviours, not least as a negative impact of self-isolating (e.g., avoidance of self-isolation to avoid emotional impact). For example, we found that students worried about how they would cope if they had to self-isolate, and experience high anxiety, low mood, and loneliness when self-isolating, coupled with a fear of re-experiencing these negative emotions if they were asked to self-isolate again. They also exhibited a strong sense of guilt if household members had to self-isolate because of them and fear the interpersonal conflict this situation might bring. Participants in our study believed that this might be a factor for young adults in decision-making related to COVID-19 testing, particularly for those who are asymptomatic. Students’ emotions seem to override their willingness to engage in COVID-19 testing when they are asymptomatic, due to the risk of self-isolation for themselves and others, despite viewing onsite testing as convenient, and seeing testing as an important national and local strategy for controlling the virus. The same pattern occurs with other protective behaviours, since people socialize to avoid feeling lonely, and loneliness is a barrier to social distancing adherence in adult populations [54]. Further, young adults are more likely to report loneliness during COVID-19 restrictive measures than other age groups [55].

Overall, our findings are consistent with others suggesting that mental health is a key driver in both testing behaviour [4], and adherence to COVID-19 protective behaviours [34]. Further exploration of students’ mental health impacts and support needs is warranted.

### 4.5. Diversity and Inclusion

Our participants proposed that the mental health impacts of social distancing and self-isolation differed between student groups. These were most notable for newly arriving students who registered at the University in October 2020, during the second surge of COVID-19 in the UK and were living in University accommodation. This was likely to be associated with a lack of social networks; these (primarily) young people had not yet established local support networks, yet social support predicts mental health and quality of life in university students [56].

The disproportionate impact of COVID-19 on young people [10,24], not only highlights a need for targeted communications to younger populations more broadly, but demonstrates the significance of structural barriers in adherence to public health messages, and the potential value of segmenting audiences for messaging to avoid making generalizations about behaviours and circumstances of particular groups [34] (such as university students). For communications in a higher education context, ‘one-size-does-not-fit-all’ and as we observed, some groups of students might feel forgotten. For example, working students (e.g., often international students, self-funded students, students with caregiving responsibilities) might have experienced a loss of income as a result of the COVID-19 related lockdown restrictions, leading to further worry, spiraling debt, uncertainty about the future, and risk of ‘falling through the cracks in the system’, all impacting on mental health [57]. The UK government ‘COVID-19 Mental Health and Wellbeing Surveillance Report’ shows that marginalised or disadvantaged groups might be disproportionally affected by the wider implications of the pandemic [58]. COVID-19 had an impact on equity and inclusion in educational settings, with young people from diverse backgrounds being at greater risk of increased vulnerability and less likely to receive the support and extra services they need [59]. Further research might be needed to explore the experiences and support needs of particular groups known to be at risk for mental health concerns, such as students with financial hardship, LGBTQI+ students, and students with special educational needs.

### 4.6. Study Strengths

Whilst vaccination levels at the time of the study were still insufficient to control population-level transmission, mass asymptomatic testing remained a prominent candidate for controlling transmission in educational settings, against the background of significant community prevalence of SARS-CoV-2 infection. Given the discovery of new variants that might be more transmissible [60], and therefore require more efficient control measures (including B.1.1.7), understanding experiences of testing and protective social behaviours in young people in schools, colleges, and universities is particularly relevant. This study sits in the context of a national debate around the implementation of mass asymptomatic testing programmes in schools and universities, which is divisive [18,19,61,62,63,64,65]. England’s Department for Education advocates weekly testing in educational settings from January 2021 [66], and despite the potential for transmission from students to other members of the community, there is little evidence of how students interpret and respond to these approaches, and the impacts of mass testing on social behaviour and wellbeing. This study therefore contributes [67] to the wider debate around mass testing and informs mitigation and containment strategies for COVID-19 in educational settings.

Remotely conducted focus group interviews were a suitable approach for exploring commonality and differences in attitudes and experiences of university students, in the context of rapidly changing national policy. Due to the crisis situation, this rapid approach allowed for early sharing of qualitative findings, which was identified as important during complex health emergencies (e.g., Ebola [68]). Early study findings were provided to the Department for Education in England and used in real-time to support institutional efforts to engage students, public health, and behavioural experts in COVID-19 messaging content and approaches to communication with students and staff. Finally, the sample included students who lived in university residences, and those who had tested for SARS-CoV-2, either at the university or via local government public health services.

### 4.7. Study Limitations and Considerations

Due to the timescale, we were unable to triangulate findings with all participants, although we confirmed themes with two participants. Students who had taken a test as part of the participating university deployment of asymptomatic testing in university residences were under-represented. Further research is needed to fully ascertain the views and experiences of marginalised groups to ensure supportive services are equitable. While students in our study were willing to express concerns in this focus group setting and talk about other students’ behaviour or compliance to COVID-19 restrictions, there might be some reservations about openly discussing any personal breaches of COVID-19 guidelines, especially given that the focus group moderators were University employees. These data relate to the views of students in a higher education setting, which might vary from that of the general public in terms of personal and attitudinal variables [69]. The frequencies presented in Table 2 do not reflect the contextual or behavioural factors that were considered during analysis, such as nonverbal cues (i.e., nodding) or participant agreement with the statements provided by others. Therefore, the number of students agreeing with the statement contributing towards each theme is likely to be underestimated. Finally, it should be noted that data were collected when the participating institution had one of the highest rates of COVID-19 in the country, although by December 2020, this had dropped below the national average for cases per 100,000 population.

### 4.8. Summary and Future Recommendations

The key findings and recommendations for practice and policy that emerged from our data are presented in Table 3.

## 5. Conclusions

Mental health of students is significantly impacted by the COVID-19 pandemic, and social isolation is a key factor in this. Fear of self-isolation is likely to influence uptake of asymptomatic testing and adherence to social restrictions, due to anxiety, guilt and low mood experienced during self-isolation. The adequacy of practical, social and emotional support for students will be paramount to encourage adherence to self-isolation, and ultimately reduce virus transmission in pandemics. Loneliness in students could be mitigated through increased intra-university communications and a focus on establishment of low transmission-risk social activities to help students build and enhance their social support networks. University communications around outbreaks and mental health support need to be timely and inclusive to reach groups of students that currently feel marginalised and are at risk of ‘falling through the cracks’ in the system. The practical and emotional support needs of students who have to self-isolate during a pandemic need to be determined, and this has relevance for other educational settings globally, particularly those in which mass testing might be implemented. Worldwide, universities need to prepare for the long-term impacts of the pandemic on student mental health and support services.

## Figures and Tables

**Table 1:** Characteristics of participants.

	Sample *n* = 25
Age (median, range)	23 (18–51)
Gender	
Male *n* (%)	9 (36)
Female *n* (%)	16 (64)
Student status ^†^	
Home students *n* (%)	19 (76)
International students *n* (%)	6 (24)
Accommodation type	
On-campus *n* (%)	8 (32)
Off-campus *n* (%)	17 (68)
Testing status ^a^	
Not tested *n* (%)	13 (52)
Symptomatic testing *n* (%)	11 (44)
Asymptomatic testing *n* (%)	1 (4)
Previously self-isolated ^b^	
Yes *n* (%)	18 (72)
No *n* (%)	7 (28)

Note: ^†^ International student; ^a^ Not tested for SARS-CoV-2, Tested Asymptomatic (University Asymptomatic Testing Service), Tested Symptomatic (NHS Symptomatic Community Testing); ^b^ Self-isolated for any reason.

**Table 2:** Examples of key themes, subthemes, frequency and their representative quotes.

Themes	Subthemes	Frequency *n* (%) *	Representative Quotes
Term-time experiences	Logistical difficulties	7 (28)	‘*For me, it was very much a case I was supposed to be going abroad this year for like a study year abroad and that was cancelled quite late notice with the Covid stuff, it was cancelled in about June/July. And so I didn’t really have much time to think about it before I had to start thinking about, you know, planning for next year and getting the practicalities sorted. So it was kind of almost a rush because I had in my head that I was going to go abroad and sorting my accommodation out there and everything like that, transport—and then suddenly everything changed and I knew I only had a couple of months to get everything together*’; Participant 12 ‘*A lot of the flats that have been in quarantine, the students have actually been forced to come out of quarantine just to get their food, because we don’t get meals or anything with our accommodation*’; Participant 15 ‘*As an international student we wouldn’t have to isolate in a usual time but during this time when we arrived at the UK we needed to isolate for two weeks first from certain countries and that happened with everyone. It’s very difficult because when you just move into a new country and you cannot do anything and you’re wondering ‘how am I going to get groceries?*’; Participant 1
	Adjustment to online learning	11 (44)	‘*All our limited lectures can be done online and it’s quite nice to be able to relax and get myself into a rhythm. It isn’t as pre-determined as it used to be*’; Participant 8 ‘*I think the university’s done well to kind of quickly get it all online actually because, you know, it’s still running and that’s the most important thing and there’s nothing—I can’t think of anything more that they could be doing*’; Participant 23 ‘*I thought that I would have really interesting experiences and networking opportunities and potentially job opportunities at the end of my matriculation and I feel very frustrated by the fact that I don’t have those opportunities anymore*’; Participant 2 ‘*I’m a new student, an international student in my Master’s degree and for me it was quite difficult to get used to all the different platforms that we use for online teaching*’; Participant 11
	Safeguarding	10 (40)	‘*On campus I actually feel relatively safe because of the social distancing. I don’t know about in halls but like in teaching, especially when we have like our labs and in-person teaching, people are actually sat away from each other and we wipe down our area*’; Participant 22 ‘*Because we all have to wear masks and visors in the labs anyway, we’re quite—and we have to social distance, 1 metre plus is the closest we’re allowed to get in the labs, so in terms of actual risk of transmission we’re lower risk than halls and social areas basically*’; Participant 13 ‘*I’m primarily lab-based and my lab was shut for 5 months due to Covid, so that’s affected my studies quite a lot*’; Participant 4 ‘*Because of the fact that my hall has a courtyard and there was a security guard watching us, it felt a bit prison-y*’; Participant 19
	Connectedness through communication	4 (16)	‘*I was actually really humbled to have an email from [my School] just to check up on me as they heard I was isolating, and that was really nice. It made me feel less forgotten*’; Participant 18 ‘*Also as a postgrad I also feel a bit forgotten about because like we were here the whole time when our labs were closed and just like a lot of the emphasis—I know we’re like a minority obviously and you can’t sort out everything at once, but it felt like a lot of the emphasis was on like majority groups that were probably less affected*’; Participant 4 ‘*I feel like they need to be in constant contact with people that are isolating or even just anyone that could make themselves known to the uni. Or just something to, I dunno, I think the uni needs to be really, really proactive in offering lots and lots of online things, constantly like daily or every evening, because I think the main thing is making sure people don’t feel alone at all*’; Participant 6
Risk perception and worry	Previous experience with COVID-19	14 (56)	‘*A guy who lives, who I share a bathroom with, tested positive but he didn’t have any symptoms so it’s been like something happening but not really anything to do with me*’; Participant 19 ‘*My mum was super bedbound for the whole ten days, but we were both very lucky, we didn’t have to go to hospital or anything. So at the beginning I wasn’t very worried about it until I kind of got it*’; Participant 6
	Perception of health	9 (36)	‘*I had no real worry for myself because, I mean, I’ve had it now so hopefully it means I’ve got some sort of immunity. I’m more worried for my older relatives*’; Participant 14 ‘*Covid terrifies me—Until recently I was considered vulnerable to the virus because of previous serious illness. So being in a shared house, still going to work in order to pay rent and also having to go onto campus for some lessons has made my anxiety go crazy*’; Participant 18 ‘*I was actually pretty excited to come back to uni, just because I know young people at the moment are having loads of cases and everything, but it kind of separates me from my parents. I know I wouldn’t put them at risk because I wouldn’t be living with them so even if I contracted the virus on campus or whether in the city of Nottingham, I would just self-isolate by myself or with my friends. I wouldn’t be putting my parents at risk. So in that sense I was kind of excited to come back and like kind of separate myself from parents*’; Participant 22
Engagement in protective behaviours	Format of communication and guidance	12 (48)	‘*I know we’ve been receiving lots of emails about what the rules are, what we need to do—but they are very text heavy—and I wouldn’t have thought of this if it weren’t for my housemates—but they’re all international students and they struggle with the large blocks of text because there are a lot of words in there that are just unfamiliar to them*’; Participant 15 ‘*There was no guidance from the government or the university that we could find about what to do when someone did test positive, so we didn’t know if we should make them stay in their room or wear a mask. We weren’t really sure*’; Participant 9 ‘*We didn’t get told that we weren’t allowed to use our communal space until after we finished self-isolating so, for us, there was no communication and then they emailed us to say ‘oh, even if you’ve all tested positive, you’re not allowed to use your communal space, or you are but one at a time’—we didn’t realise this—so that seems like really weird because, to be fair, if you’ve tested positive it’s probably too late and you’ve probably given it to all your flatmates anyway*’; Participant 14 ‘*And the only place it said what the mealtimes were initially for the first couple of weeks was on this email that we got and there’s now a poster once you’re at the food counter telling you what times you should be there. I think at that point it’s a bit too late if you’ve turned up at the wrong time*’; Participant 5
	Environmental and structural factors	9 (36)	‘*There were just like large crowds of them in the corridor over both sides of the system and then we would all get like stuck in crowds of students*’; Participant 4 ‘*I feel like the majority of people are self-isolating, at least here, and it got a lot better now they’ve got the categories in, because before they were trying to deliver to everyone who was isolating, which there were way too many people for that to work, so now that if you don’t have symptoms but are isolating you’re allowed to go to the dining room, it works so much better. And also you get to actually see people, which makes it feel a bit better, even if you’re in different households you’re not allowed to sit near them, you still get out your room*’; Participant 16
	Desire for social contact	12 (48)	‘*It’s the fact that people want to socialise more than they’re worried about the rules*’; Participant 19 ‘*I think also people will social distance with strangers or people they don’t know, but they feel it’s fine with friends, even if they’re not in the same household, which can be hard because literally some people, like the only friends they have are not in their household and now it’s dark, it’s getting cold and it’s like sit in your room alone or, like, break the rules, and especially now you’re not allowed people in your household even if you’re social distancing with them, I reckon lots of people are going to not follow that*’; Participant 16
Openness to testing	Control of the virus	6 (24)	‘*There clearly are people who are asymptomatic but carrying the virus and being able to get on top of that is going to play a massive role in being able to control the virus*’; Participant 13 ‘*I think it’s a good idea in terms of—because obviously the whole point of wider testing means you’ve got a better ability to potentially control the virus and that’s, like, in all other countries that have done good control and lots of testing is seen as a good thing*’; Participant 25 ‘*I fully agree with halls being prioritised because you’re kind of mixing without PPE and stuff—but I know from the amount of work that’s going in to trying to get postgrad researcher back in labs, I can imagine the asymptomatic screening would be really useful if there were capacity to do it*’; Participant 13 ‘*My only sort of slight issue with the asymptomatic testing is it does—it kind of puts, like, it makes [City] seem a lot worse than potentially it actually is in comparison to other areas of the country and other universities*’; Participant 5
	Access and experience	8 (32)	‘*I think it’s quite a good thing because otherwise you can’t really have a test unless you’ve got symptoms*’; Participant 19 ‘*I think it’s pretty much impossible to get an NHS test unless you’ve got really loads of symptoms and even if you do it’s still really a long wait and you have to like drive somewhere. Like we see it on BBC news every single day how they’re all backed up. So I think in a way it’s quite nice to see. I know it’s not everyone that gets the best but at least some halls and like quite a lot of people are getting tested, which is quite good, that otherwise wouldn’t have been tested*’; Participant 22 ‘*Like it’s not terrible but it’s just a bit uncomfortable but it was quite easy to do, just like setting up that appointment online and then just going to the walk-in and it was a bit weird because it was that big white tent and it felt very much like a big Hollywood film or something. It wasn’t too bad because everyone was really friendly and really helpful. I found it quite—even though it wasn’t a massively fun experience, it was kind of a positive experience*’; Participant 6 ‘*I was impressed, I had my NHS results back within 2 days. The test was easy and quick*’; Participant 18
	Perceived immunity	6 (24)	‘*I’ve had a few friends who’ve mentioned that they do want to get tested positive because they’re quite confident on their immunity and so they’re just like ‘I want to get tested positive so that I can feel free about social distancing and not really have to feel so restricted*’; Participant 11 ‘*I’d be quite wary around some of the people that I know. I sort of fear that once they’ve got it they’re going to feel like they’re immune and they can do whatever now. I’ve certainly seen like parties of households as soon as they come out of isolation, they sort of celebrate and go a bit mad*’; Participant 10
Barriers to testing	Guilt about impact of test result on others	6 (24)	‘*I feel people feel guilty if they have it and then that means everyone in the household has to isolate and then, like, the prospect of having to isolate in a pretty small room for like two weeks is quite daunting as well*’; Participant 16 ‘*I think once you’re living in a bigger household, the guilt of knowing you’re going to make everyone have to isolate with you, would be a very strong detractor*’; Participant 10 ‘*In our house—we’re kind of getting it sorted now—but unfortunately we had some tensions over, like, to what extent the regulations hold specifically—and I’ve asked a few people their opinions of this—it’s like there’s one person that tests positive, should they be, like, literally allowed to leave their room*’; Participant 24
	Mental health impact of testing	8 (32)	‘*Some of my friends don’t want to get tested because if they are positive they have to isolate and they’re really scared of the loneliness, kind of thing*’; Participant 4 ‘*I think it’s that point about lockdown that really got me because, again, over the summer, especially that sort of first half of the summer, my mental health just completely deteriorated and went like really, really badly and I’m in the position now and sort of getting a positive test is I don’t want to go back to what that was like, being locked down*’; Participant 24
General wellbeing	Social impact of the pandemic	10 (40)	‘*I think that’s going to be the tough thing for students, is that sort of emotional wellness boost that we all get from being around other people*’; Participant 15 ‘*I’d say I feel very strongly about the first years and sort of hearing, like, about, like, where they get like a positive test in a hall, they’ve got security guards on the door and they’re sort of, like, breaking the law because they want to go and see their mates and I remember how difficult it was in first year, like, to meet people, to make friends—and when you don’t have those obvious, like, big social weeks to meet people and the university is, like, encouraging them to not go out and meet people—I can’t imagine how difficult that is for some people*’; Participant 25 ‘*I would say as a fresher starting, it’s been really hard to actually meet people and I think what a lot of people are worried about is the fact that, you know, in the next couple of months we’re going to have to choose who we want to live with next year and maybe even for like the rest of our course and we’ve really not had an opportunity to meet people outside of an academic setting*’; Participant 5
	Mental health impact of the pandemic	8 (32)	‘*I’m getting quite down about it, because it’s literally your work’s on your screen, like, and then all you can do is like looking at your screen, like, if you socialise you have to do it over on your screen and it’s just really, just making me a bit down really, because I can’t even eat lunch with people*’; Participant 16 ‘*Nationally if we went into another lockdown I would be scared about how I coped because I coped pretty badly in the last one with my mental health and stuff and I’d just be scared I’d go straight back into that again if that were to happen*’; Participant 23

* Number of participants who contributed independent statements towards each theme. These figures do not reflect contextual or behavioural factors, such as nodding in agreement or participant agreement with the statements provided by others. PPE = personal protective equipment; and NHS = UK National Health Service.

**Table 3:** Key points and policy recommendations.

**Practical impacts during Autumn return to campus** Last minute changes to accommodation, travel plans and academic timetabling. Challenges of accessing basic supplies and help with everyday living. Shift to online learning modality. Pandemic impacts on academic studies (e.g., halted laboratory work and research). Greater impacts for those without social supports and social networks.
**Emotional impacts during Autumn return to campus** Fear, worry, anxiety, guilt, low mood are widespread. Some reports of food insecurity. However, students do not feel unsafe being at university during the pandemic.
**Risk perceptions** Those with prior experience of COVID-19 (virus/self-isolation) feel more at risk. Vulnerable groups (pre-existing conditions) feel more at risk. Most students worry more about risks to others than themselves.
**Engagement in protective behaviours (social distancing, self-isolation)** Timeliness of communications will influence behaviour. Presentation of communications is important—‘one-size-does-not-fit-all’. Environmental and structural factors play a role in social distancing on campus. Desire for social contact is strong and can override perceived risk and regulations. Primary reason for seeking social contact/breaking self-isolation is to avoid or mitigate the emotional impacts of social isolation.
**Mass asymptomatic testing on campus** Students are receptive to mass asymptomatic testing. Testing is seen as a mechanism for getting control over the virus. Availability of testing on campus enhances students’ perceptions of safety. Reports of convenience, accessibility and positive experience around testing. Most students would adhere to social behaviour guidelines whether the test result is +ve or –ve. Risk of ‘perceived immunity’ and breaking self-isolation rules but only in a minority. Barriers to testing are primarily emotional factors associated with self-isolation (e.g., guilt about the impact of self-isolation on others, and fear of the mental health impact of self-isolation).
**Broader and longer-term impacts of COVID-19** This pandemic will have long-term impacts on student experience and satisfaction. Coping with social isolation is harder for students without established social networks. Social contact is intrinsically tied to students’ emotional wellbeing. Some students fear for the future, and many have sustained mental health concerns that will need to be addressed.
**Recommendations** Practical and emotional impacts of a pandemic are significant and need to be accounted for when assessing student engagement in studies and academic progress. Action plans are needed to ensure equitable mobilisation of basic supplies for students living on and off campus, in the face of another pandemic. Clear statements are required on expectations of student behaviour. Guidance on pandemic-related social behaviour and testing needs to be regular, rapid and inclusive—‘one size does not fit all’ for messaging. Providers should consider incentives for compliance, and disincentives for non-compliance such as the use of disciplinary measures in serious cases. Implementation of mass testing programmes requires significant support in place for students who might be required to self-isolate to minimise the risk of virus transmission. Practical, social and emotional support needs of self-isolating students should therefore be identified and should take into account the needs of marginalised groups. Supportive services should seek to enhance social connectedness, inclusion and positive mental wellbeing. Universities need to prepare for the longer-term impact of pandemic-related mental ill-health on support and welfare services.

## Data Availability

The data presented in this study are available on request from the corresponding author. The data are not publicly available due to risk of participant identification.

## Supplementary Materials

The following are available online at https://www.mdpi.com/article/10.3390/ijerph18084182/s1. File S1: Tier 2 High Risk Alert National Restrictions; File S2: Consolidated criteria for reporting qualitative studies (COREQ): 32-item checklist, File S3: Individual characteristics of participants; File S4: Focus Group Question Guide; and File S5: Thematic map illustrating the relationships between the key themes and subthemes.
