# Supplementary material for: Students’ Views towards Sars-Cov-2 Mass Asymptomatic Testing, Social Distancing and Self-Isolation in a University Setting during the COVID-19 Pandemic: A Qualitative Study
Source: Int J Environ Res Public Health. 2021 Apr 15;18(8):4182. doi: 10.3390/ijerph18084182 (PMC8071290; doi:10.3390/ijerph18084182)
Supplement: Supplementary file 1 [file ijerph-18-04182-s001.zip › Supplementary File 1.pdf]

Supplementary File 1: Tier 2 High Risk Alert National Restrictions

The 5-Tier System in England
The lowest tier (1: medium risk alert) replicates the existing restrictions that apply nationwide by default. The upper tiers at the time of the study (2: high and 3: very high risk) standardised local restrictions for regions with higher rates of infection, with two additional top-level tiers (4: regional stay at home and 5: national lockdown) introduced in December 2020 and January 2021 respectively. At the time of the study, the alert level was being reviewed every 14 days and the rules and regulations were reviewed every 28 days.

Tier 2 – October 2020
Criteria for Tier 2 High Risk Alert National Restrictions were in place in England during October 2020 and were outlined to the general public here: https://www.gov.uk/coronavirus (accessed on 30.10.2020)

Meeting with others: You can see people from different households outside in groups of up to 6 people but you can only meet inside with those in your household or support bubble.

Travel and transport: Journeys should be limited where possible, but you can still travel and use transport to go to the shops, work and hospitality venues that are open. You should still wear a face covering.

Staying overnight: You can only stay overnight somewhere if it's with those in your household or support bubble.

Going to work: You should work from home where possible. Where this isn't possible, workplaces should be coronavirus secure.

Shops: These can remain open.

Hospitality: Restaurants, pubs, cafés and other hospitality venues remain open. These venues must close by 10pm and provide table service. You can only go out to these places with people from your household or support bubble, unless you're outside and in a group of no more than 6 people.

Exercise and sporting: Gyms can remain open. Organised sport and licensed physical activity are allowed in indoor and outdoor settings but may be subject to certain rules. You can't attend sporting events such as football matches.

Places of worship: They can open as long as households don't mix indoors, but it's best to check with your place of worship. There are exceptions for weddings and funerals.

Weddings: Up to 15 people can attend a ceremony and a coronavirus secure sit-down reception.

Funerals: Up to 30 people can attend someone's funeral and up to 15 people can attend someone's wake, but this can't be held in someone's home.

Care homes: It's unlikely you'll be able to visit a loved one at this time, except in exceptional circumstances, such as if someone is coming to the end of their life. Contact the care home for more information as every care home has a different policy.

Public buildings such as libraries: These can remain open.
