# Supplementary material for: Students’ Views towards Sars-Cov-2 Mass Asymptomatic Testing, Social Distancing and Self-Isolation in a University Setting during the COVID-19 Pandemic: A Qualitative Study
Source: Int J Environ Res Public Health. 2021 Apr 15;18(8):4182. doi: 10.3390/ijerph18084182 (PMC8071290; doi:10.3390/ijerph18084182)
Supplement: Supplementary file 1 [file ijerph-18-04182-s001.zip › Supplementary File 2.pdf]

Supplementary File 2. Consolidated criteria for reporting qualitative studies (COREQ): 32-item checklist

Topic and Item No.	Guide Questions/Description	Response
**Domain 1: Research team and reflexivity**		
Personal Characteristics		
1. Interviewer/facilitator	Which author/s conducted the focus groups?	Holly Blake (HB) and Holly Knight (HK)
2. Credentials	What were the researcher's credentials? E.g. PhD, MD	HB: PhD, CPsychol.<br>HK: PhD, CPsychol
3. Occupation	What was their occupation at the time of the study?	HB: health psychologist/university academic;<br>HK: clinical psychology researcher
4. Gender	Was the researcher male or female?	2 female
5. Experience and training	What experience or training did the researcher have?	HB and HK: Senior mixed-methods researchers, experienced interviewer and focus group moderator, GCP trained
Relationship with participants		
6. Relationship established	Was a relationship established prior to study commencement?	Researchers met the participants during recruitment
7. Participant knowledge of the interviewer	What did the participants know about the researcher? e.g. personal goals, reasons for doing the research	Participants knew that HB was a university academic health researcher, and that HK was a health researcher. They knew that both interviewers were affiliated to the participating university.
8. Interviewer characteristics	What characteristics were reported about the interviewer/facilitator? e.g. Bias, assumptions, reasons and interests in the research topic	Participants knew that HB and HK were interested in the experiences of students during the COVID-19 pandemic.
**Domain 2: Study design**		
Theoretical framework		
9. Methodological orientation and Theory	What methodological orientation was stated to underpin the study? e.g. grounded theory, discourse analysis, ethnography, phenomenology, content analysis	Thematic analysis
Participant selection		
10. Sampling	How were participants selected? e.g. purposive, convenience, consecutive, snowball	Purposive (e.g. gender, undergraduate or postgraduate, living circumstances (on/off campus), self-isolated or not, had a COVID-test or not)

11. Method of approach	How were participants approached? e.g. face-to-face, telephone, mail, email	Participants were approached and recruited by email
12. Sample size	How many participants were in the study?	25 participants took part in qualitative focus groups
13. Non-participation	How many people refused to participate or dropped out? Reasons?	1 student signed up to a focus group but did not attend due to other commitments.
Setting		
14. Setting of data collection	Where was the data collected? e.g. home, clinic, workplace	Data were collected online (video-conferencing platform)
15. Presence of non-participants	Was anyone else present besides the participants and researchers?	No.
16. Description of sample	What are the important characteristics of the sample? e.g. demographic data, date	Students: self-isolated/not, COVID-tested/not, living circumstances (on/off campus).
Data collection		
17. Interview guide	Were questions, prompts, guides provided by the authors? Was it pilot tested?	Questioning guide used for each focus group. Items were pilot tested with students in a patient and public involvement and engagement (PPIE) group.
18. Repeat interviews	Were repeat interviews carried out? If yes, how many?	No. A total of 4 focus groups were conducted.
19. Audio/visual recording	Did the research use audio or visual recording to collect the data?	Focus groups were audio-recorded using a video-conferencing platform. Interviewers kept cameras on to assist with establishing rapport.
20. Field notes	Were field notes made during and/or after the interview or focus group?	Yes.
21. Duration	What was the duration of the interviews or focus group?	Approx. 60-90 mins.
22. Data saturation	Was data saturation discussed?	Yes.
23. Transcripts returned	Were transcripts returned to participants for comment and/or correction?	No – due to time sensitivity of the research
**Domain 3: analysis and findings**		
Data analysis		
24. Number of data coders	How many data coders coded the data?	Two.
25. Description of the coding tree	Did authors provide a description of the coding tree?	No, however initial coding was informed by the interview

		guide, and coding was continuously refined.
26. Derivation of themes	Were themes identified in advance or derived from the data?	Themes were derived from the data.
27. Software	What software, if applicable, was used to manage the data?	No software was used; data was cut and pasted and grouped and colour coded in a word document and excel spread sheet.
28. Participant checking	Did participants provide feedback on the findings?	Yes.
Reporting		
29. Quotations presented	Were participant quotations presented to illustrate the themes / findings? Was each quotation identified? e.g. participant number	Yes.
30. Data and findings consistent	Was there consistency between the data presented and the findings?	Yes.
31. Clarity of major themes	Were major themes clearly presented in the findings?	Yes.
32. Clarity of minor themes	Is there a description of diverse cases or discussion of minor themes?	Yes.
