# Supplementary material for: Students’ Views towards Sars-Cov-2 Mass Asymptomatic Testing, Social Distancing and Self-Isolation in a University Setting during the COVID-19 Pandemic: A Qualitative Study
Source: Int J Environ Res Public Health. 2021 Apr 15;18(8):4182. doi: 10.3390/ijerph18084182 (PMC8071290; doi:10.3390/ijerph18084182)
Supplement: Supplementary file 1 [file ijerph-18-04182-s001.zip › Supplementary File 3.pdf]

Supplementary File 3: Individual characteristics of participants

ID (Participant)	Age	Gender	Home/Int†	On/Off campus	Status[a]	Self-isolated[b] /Not
1	22	F	International	Off campus	Not tested	Yes
2	51	F	International	Off campus	Not tested	Yes
3	20	M	Home	Off campus	Not tested	No
4	25	F	Home	Off campus	Not tested	No
5	25	F	Home	On campus	Not tested	No
6	24	F	Home	Off campus	Sympt	Yes
7	32	M	International	Off campus	Sympt	Yes
8	20	M	Home	Off campus	Sympt	Yes
9	20	M	Home	Off campus	Sympt	Yes
10	25	F	International	On campus	Not tested	No
11	22	F	International	Off campus	Not tested	Yes
12	20	F	Home	Off campus	Not tested	Yes
13	32	F	Home	Off campus	Sympt	Yes
14	18	M	Home	On campus	Sympt	Yes
15	24	F	Home	On campus	Sympt	Yes
16	18	F	Home	On campus	Asympt	Yes
17	20	F	Home	Off campus	Sympt	Yes
18	20	F	Home	Off campus	Sympt	Yes
19	18	M	Home	On campus	Not tested	No
20	18	F	Home	Off campus	Not tested	Yes
21	21	M	International	On campus	Not tested	Yes
22	20	F	Home	On campus	Not tested	No
23	22	M	Home	Off campus	Sympt	No
24	20	F	Home	Off campus	Not tested	Yes
25	20	M	Home	Off campus	Sympt	Yes

Note: [†]International student; [a]Not tested for SARS-CoV-2, Tested Asympt. (University Asymptomatic Testing Service), Tested Sympt. (NHS Symptomatic Community Testing); [b]Self-isolated for any reason.
