# Supplementary material for: Students’ Views towards Sars-Cov-2 Mass Asymptomatic Testing, Social Distancing and Self-Isolation in a University Setting during the COVID-19 Pandemic: A Qualitative Study
Source: Int J Environ Res Public Health. 2021 Apr 15;18(8):4182. doi: 10.3390/ijerph18084182 (PMC8071290; doi:10.3390/ijerph18084182)
Supplement: Supplementary file 1 [file ijerph-18-04182-s001.zip › Supplementary File 4.pdf]

Supplementary File 4: Focus Group Question Guide

1. Tell us a little about yourself (Interview Sample Characteristics)
[age, undergraduate or postgraduate, year of study, what you study, international or home student, living at home, halls, private accommodation (on or off campus), whether they have taken part in the university testing programme or had a COVID19 test elsewhere]

2. Views about COVID19
What are your views about COVID19? Do you have any particular *experiences with COVID*, concerns or worries?

3. Views on university life during COVID19

(a) *For those who are returning students*: How did you feel about returning to the university/campus during this time? What are some reasons for returning/ did you feel you had a choice? Are you coping with the changes to campus life? Is anything particularly difficult/what have you found helpful in coping with these changes?

(b) *For those who are new students*: How did you feel about starting University during this time? Discuss some reasons for starting. Are you coping? Is anything particularly difficult/what have you found helpful in coping with the transition to university?

(c) There is a lot of *media attention* being paid to universities at the moment, what are your thoughts on this?

4. Views towards actions to protect people from COVID19

(a) *Protective behaviours and adherence*: What do you think students are typically doing with relation to guidance around handwashing, face coverings, and social distancing?

(b) *Social distancing*: What are your own views on social distancing? Is it important to you as an individual or not? Why? What do you think is the main value of social distancing in a university setting? What benefits might it have? What are the main influences on the choices students make around social distancing? What do you think will be the main challenges for students in adhering to social distancing guidelines?

(c) *Self-Isolation*: What are your views on self-isolation? Is it important, or not? (being asked to stay at home for a period of time if you have been in contact with someone who has COVID, or have symptoms or a positive test yourself). Do you think that students will self-isolate if they are asked to do so? What would encourage students to self-isolate if they were asked to? How can we best support them during periods of self-isolation? What would prevent them, or discourage them from adhering to self-isolation advice?

5. Views on COVID testing

(a) *What are your views on asymptomatic COVID testing in universities*? [The university is running a COVID testing programme which includes asymptomatic testing in halls of residence - testing people even if they don't have symptoms]. Do you think university COVID testing will change how students feel in any way?

(b) *Have you taken a COVID19 test yourself?* Was this as part of the university testing programme, or was it an NHS test, or both? How was this process? What went well? Were there any particular issues or problems? How did you feel when you got your result? (disclosing result is optional) Or – how do you think students will feel if they get a negative or positive test result?
