# Supplementary material for: Students’ Views towards Sars-Cov-2 Mass Asymptomatic Testing, Social Distancing and Self-Isolation in a University Setting during the COVID-19 Pandemic: A Qualitative Study
Source: Int J Environ Res Public Health. 2021 Apr 15;18(8):4182. doi: 10.3390/ijerph18084182 (PMC8071290; doi:10.3390/ijerph18084182)

Supplementary File 5. Thematic map illustrating the relationships between the key themes and subthemes

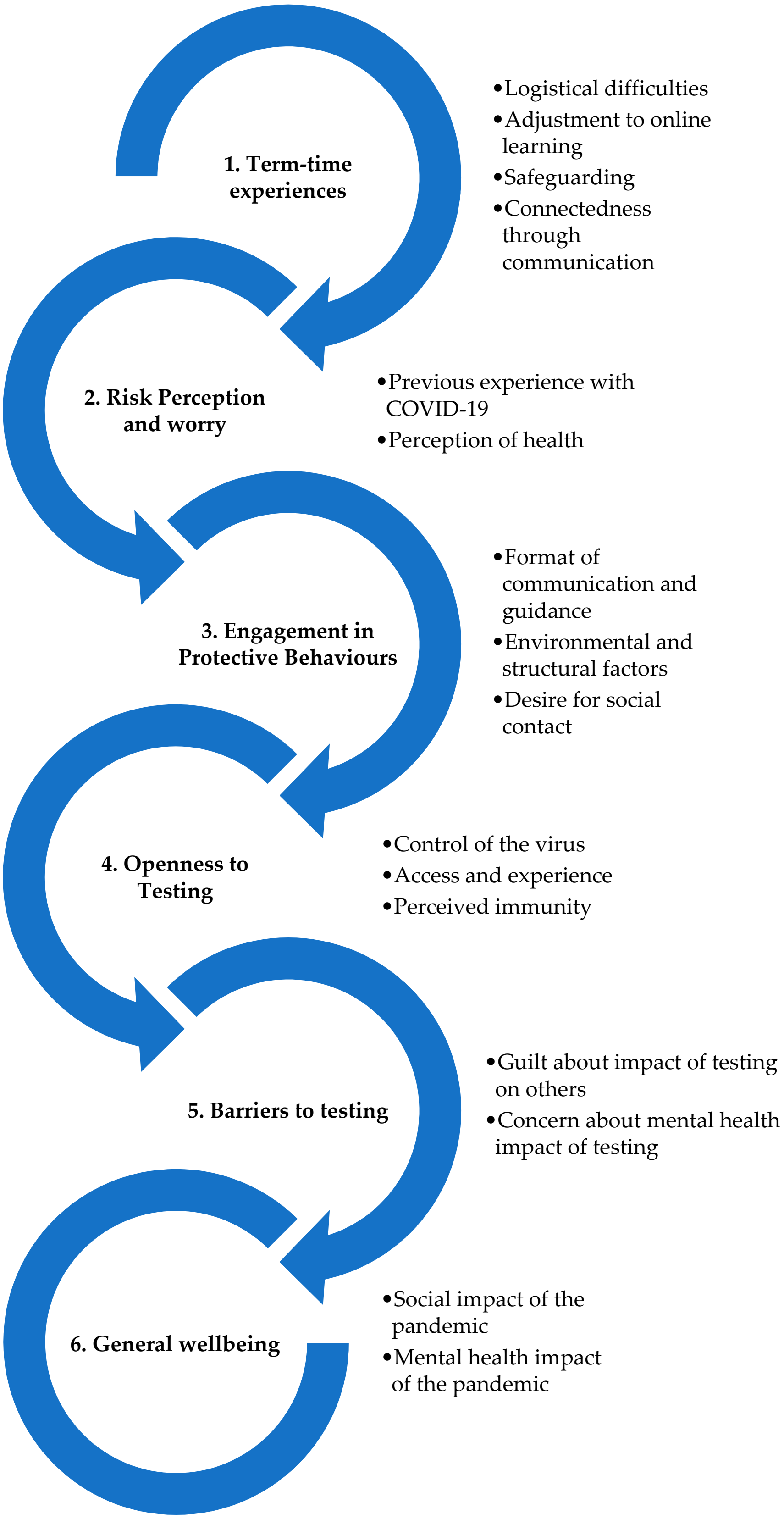

Supplement: Supplementary file 1 [file ijerph-18-04182-s001.zip › Supplementary File 5.pdf]
